# Is not workplace gossip bad? The effect of positive workplace gossip on employee innovative behavior

**DOI:** 10.3389/fpsyg.2022.1017202

**Published:** 2022-11-24

**Authors:** Yuping Dai, Xiangzhi Zhuo, Jie Hou, Bei Lyu

**Affiliations:** ^1^Faculty of Business, City University of Macau, Macau, China; ^2^School of Economics and Management, Huaibei Normal University, Huaibei, Anhui Province, China; ^3^School of Foreign Languages, Huaibei Normal University, Huaibei, Anhui Province, China; ^4^Chinese Graduate School, Panyapiwat Institute of Management, Nonthaburi, Thailand

**Keywords:** positive workplace gossip, employees’ innovative behavior, employee loyalty, organizational trust, Chinese context

## Abstract

**Purpose:**

The purpose of this study is to examine the role of positive workplace gossip (PWG) in employee innovative behavior, whereby a mediating effect of employee loyalty is proposed in this relationship. The moderating effect of organizational trust (OT) is also examined on the indirect of PWG on employee innovative behavior through employee loyalty.

**Design/methodology/approach:**

This research used a survey data of 327 employees from the enterprises selected from the Pearl River and Yangtze River Delta region of China. Based on the literature review, five main hypotheses were formulated and explored. The SPSS-Process Macro Plugin was used to analyze the hypothesized model.

**Findings:**

Results show there is a positive and significant relationship between PWG and employee innovative behavior. This study also confirm that employee loyalty is an intervening variable and OT as a moderator.

**Practical implications:**

Organizations should pay more attention to workplace gossip phenomena, encourage employees to take appropriate part in positive workplace gossip and to communicate positive information about other colleagues, and build an inclusive, open, sincere, and interdependent platform in the organization.

**Originality/value:**

Employee innovative behavior plays an essential role in organization’s survival and development. Few studies have investigated PWG may promote employee innovative behavior through employee loyalty. The data, model, and findings of this research address the gap and complement the current state of knowledge.

## Introduction

Gossip is almost unavoidable in the real-world social network (Grosser et al., 2012; Eckhaus et al., 2019), deemed as a far-reaching informal channel of information exchange aside from the communication of formal information within the organization (Wu et al., 2018; Dores Cruz et al., 2021). Workplace gossip is defined as the informal, evaluative discussion about the colleagues or leaders who are absent (Ellwardt et al., 2012c; Eckhaus et al., 2019). The subjects involved in workplace gossip include the disseminator, the listener, and the target of gossip (Foster 2004; Estévez et al., 2022). By its influential effect, workplace gossip can be classified into positive gossip (e.g., compliment of someone’s professional ability and promotion) and negative gossip (e.g., discussion about someone’s theft, bribery, lassitude at work, divorce, etc.; Ellwardt et al., 2012c; Spoelma and Hetrick 2021). Gossip is a complicated behavior. From the traditional viewpoint, gossip is harmful as it would demoralize the organization (Spoelma and Hetrick 2021), defame employees, force outstanding employees to quit, and reduce employee’s organizational citizenship behaviors (OCBs; Wu et al., 2018), leading to the emotional exhaustion of the gossip targets and impairing their creativity (Liu et al., 2020). But in the recent years, researchers have proposed that positive gossip and negative gossip are in equilibrium distribution within the organization (Spoelma and Hetrick 2021). Therefore, attention should be paid to the influence of PWG upon employee’s working behavior.

Although the existing literature on workplace gossip is extensive and focuses particularly on its “dark side” (Zhou et al., 2019; Spoelma and Hetrick 2021; Zong et al., 2021), some previous research has also noted the positive relationship between workplace gossip and employee psychology and work-related outcomes, such as exchanging of information (Ellwardt et al., 2012c), reducing social loafing (Spoelma and Hetrick 2021), providing a means of stress relief (Grosser et al., 2012), satisfying one’s curiosity, and enhancing the friendship between gossipers (Foster 2004; Brady et al., 2017).

Researchers have verified the impact of gossip on personal creativity or innovation (Zhou et al., 2019; Liu et al., 2020). However, they focused mostly on negative workplace gossip. Studies on PWG and employee innovative behavior are sparse. Employee innovative behavior is a cornerstone of corporate innovation, as well as a strong assurance for the enterprise to adapt quickly to the complicated and changeable business environment (Olokundun et al., 2021). Social contact environment is a key factor that may impede or promote individual innovation (Amabile and Pratt 2016). PWG is an ideal tool to create social contact (Brady et al., 2017), getting into certain circles and building friendship between employees (Foster 2004). Besides, innovation is an interactive process that involves communication and cooperation between different members (Østergaard et al., 2011), PWG could facilitate communication and knowledge exchange between gossipers (Ellwardt et al., 2012c; Dores Cruz et al., 2021). On the above, we predict that PWG may encourage the disseminator to exhibit more innovative behaviors in the work.

PWG could promote information and friendship bonds (Ellwardt et al., 2012b; Zong et al., 2021), bring about a harmonious atmosphere to the organization, and fulfill employee’s emotional needs. According to the social exchange theory, the interpersonal communication within an organization is in essence a series of exchanges based on “the principle of reciprocity” (Cropanzano and Mitchell 2005). When perceiving the rich instrumentality and emotional support, employees are more ready to contribute their wisdom and talents to organizational development, built a loyalty to the organization (Mossholder et al., 2005). We propose that PWG may also enhance employee innovative behavior by stimulating employee loyalty. Furthermore, trust is a lubricant for benign operation of the organization (Lewis and Weigert 1985). Trust is the key to maintaining social relations and fostering different positive working attitudes (Ugwu et al., 2014; Alshaabani et al., 2022). Grosser et al. (2010) point out that trust is a prerequisite for the spread of gossip. Therefore, we suspect that organizational trust is the boundary condition of the link between PWG and employee innovation behavior.

On the above, with Chinese enterprises as the research object, this study explored the mediating effect and boundary condition of PWG on employee innovative behavior and made up for the deficiencies in existing studies, with main contributions in the following aspects: First, given that PWG is a new research topic (Grosser et al., 2010), not only is there a lack of research on the mediating mechanism of PWG on employee innovative behavior, but the direct impact of PWG on employee innovative behavior is rarely discussed. This study would extend the research on employee innovative behavior. Second, for the recent century, anthropologist have always been observing and discussing the role of gossip in the group (Brady et al., 2017). The existing studies have provided profound insights into the antecedent variables of workplace gossip, but are limitedly focused on the consequent variables (Wu et al., 2018; Spoelma and Hetrick 2021), even rarely focused on PWG. Therefore, this study enriches the ones on the consequent variables of workplace gossip. Third, whether PWG or organizational trust is a situational factor of workplace. This study integrated both together to examine their influence upon employee innovative behavior, supplementing the studies on the influence of multiple situational factors upon employee innovative behavior.

## Literature review and hypotheses

### Positive workplace gossip on employee innovative behavior

PWG mainly involves individual fulfillment and reputation (Grosser et al., 2012; Ellwardt et al., 2012a). Sending positive gossip, such as praising or defending others, will, in turn, generate similar support from others. Social support in organizations is an effective way to promote workplace friendships and positive interpersonal relationships (Ellwardt et al., 2012a; Brady et al., 2017; Chen et al., 2020; Su et al., 2020; Zong et al., 2021; Lyu et al., 2022). Amabile and Pratt (2016) put forward that social environment is a key factor that may impede or promote individual creativity. Employee innovative behavior calls for the role of an innovation atmosphere (Hsu and Chen 2017). Hence, communicating PWG is an effective means to influence the innovation atmosphere as well as a situational source of promoting individual innovative behavior.

Besides, the social identity theory suggests that people classify themselves through social comparison and group identification and generate “in-circle” and “out-circle” consciousness. They form different attitudes and behaviors toward the groups that emotional preference for the “in-circle” and the rejection for the “out-circle” (Turner 1975). Brady et al. (2017) pointed out that PWG is a more ideal tool to build social relations than negative workplace gossip. PWG is a key measure that helps employees develop closer affinity with colleagues in the workplace and create a solidary workplace atmosphere (Noon and Delbridge 1993), and people who spread positive gossip are more likely to be liked in a group (Farley 2011). On the basis of the above analysis, we infer that communicating the positive gossip helps the gossip disseminator draw closer relationship to the gossip target and get into certain circles. When individuals feel themselves a member of the circle, a good sense of belonging is built in them, so that they are more inclined to concentrating on their own job and improving creativity. Therefore, this study proposed the following hypothesis:

*H1*: Positive workplace gossip positively affects employee’s innovative behavior.

### Employee loyalty as a mediator

Frequent employee turnover can put the company in an awkward position, such as profit cuts, and affect the work efficiency and mental state of other employees within the company (Phuong and Vinh 2020). Therefore, employee loyalty plays an important role in formulating precise organizational development policies and achieving organizational goals (Zanabazar and Jigjiddorj 2021). Some studies point out it takes a cost equivalent to 6–9 months of emoluments to recruit, train, and orient a new employee (Beehner and Blackwell 2016). Therefore, how to retain talents and foster employee loyalty has always been a focus of concern of researchers and management practitioners in modern corporate management. Employee loyalty is defined as individual identification of the organizational core value concept, which reflects the mental states of employees and the employer and the individual decision of readiness to remain working as hardest as possible at the enterprise (Meyer and Allen 1991). Employee loyalty is a centralized expression of the mind and emotions, and it intensifies with the increase in satisfaction (Dhir et al., 2020). Employee loyalty may be embodied in the readiness to work till late, low quit rate, and readiness to deliver more services for the enterprise (Guillon and Cezanne 2014).

PWG is one of the means to develop good social relations (Ellwardt et al., 2012a,b; Dores Cruz et al., 2021). Inferior interpersonal relationships might impose huge costs on the organization, including a high quit rate. PWG could promote workplace friendship (Ellwardt et al., 2012b), while the friendship can, in turn, promote the communication, and enhance loyalty, trust, and commitment (Zaheer et al., 1998). Furthermore, a good socializing environment can promote employee satisfaction with the existing working environment, which can further lower the quit rate (Dhir et al., 2020). Therefore, it can be inferred that PWG can affect employee loyalty.

Meanwhile, employee loyalty is a key predicative variable affecting employee behavior, which has received wide attentions from the academia and the practice field. Employee loyalty is representative of organization members’ attitude of identifying with the organization, behavior of continuous support, and readiness to contribute their power and pay out more extra-role behaviors for the enterprise to achieve its goals (Hui et al., 2012). High-loyalty employees have strong senses of attachment and belonging, and they are ready to improve and protect the organization, keeping a strong emotional connection with it. The stronger the emotional connection between employees and the organization, the more innovative behaviors they exhibit at work (Eisenberger and Rhoades 2001). Therefore, we proposed the following hypothesis:

*H2*: Employee loyalty acts a mediating role between PWG and employee innovative behavior.

### Organizational trust as a moderator

According to the social exchange theory, the employee-organization social exchange includes not only material exchange but also spiritual exchange, such as organizational trust. Organizational trust is a key situational variable in social exchange relations. When feeling benefited from the organization, employees would reward their organization in a positive manner. Some studies have pointed out that organizational trust is conducive to improving innovators’ organizational identification, stimulating employee’s OCBs (Verburg et al., 2018), promoting employee satisfaction and dedication, enhancing organizational affective commitment, reducing turnover intention, urging employees to foster good working attitudes and behaviors (Alshaabani et al., 2022), and encouraging employees to engage in extra-role behaviors (Singh and Srivastava 2016). Therefore, it can be speculated that employee loyalty and employee innovative behavior are subject to a large extent to organizational trust.

Trust stems from social activities (Shore et al., 2006). Although there remain no studies specialized in the relation between organizational trust and PWG thus far (Bencsik and Juhasz 2020), we can infer from previous studies that the gossip in organization is closely related with organizational trust. It is risky to circulate workplace gossip for the disseminator or for the gossip target, but this risk tends to decline in a trustful relation (Ellwardt et al., 2012c). The trust in interpersonal relationships is an important driving force to raise employee satisfaction. When trusted within the organization, employees would mentally feel themselves to be an “organization insider” and thus are more ready to share information and join discussion in the organization. In other words, workplace gossip would more likely be circulated with the rise in the level of interpersonal trust. The higher the degree of employee’s trust in the organization, the stronger the desire to communicate positive gossip, and the greater the influence of positive gossip on employee loyalty.

In addition, employee’s organizational trust stems from the organizational support perceived by employees. When feeling the support and trust from the organization due to the organization’s system, procedures, policies, and climate, employees would develop higher loyalty and professional ethics and are more ready to exhibit innovative behaviors in the work. Yu et al. (2018) pointed out that an employee working in a trustful environment would take active part in collective discussion and stimulate novel ideas instead of feeling any hostility from other employees, so that the employee innovative behavior is promoted. In other words, individuals tend to express a stronger sense of loyalty in a high organizational trust working environment and are more likely to bring forth active innovative thinking and demonstrate stronger innovative capability. Based on the above analysis, two additional hypotheses were proposed below:

*H3*: Organizational trust can positively regulate the positive effect of positive workplace gossip on employee loyalty. The higher (lower) the organizational trust, the stronger (weaker) the positive relation of positive workplace gossip to employee loyalty.

*H4*: Organizational trust can positively regulate the positive effect of employee loyalty on employee’s innovative behavior. The higher (lower) the organizational trust, the stronger (weaker) the positive relation of employee loyalty to employee’s innovative behavior.

*H2* and *H3*, *H2* and *H4* show a moderated mediation effect, that is, employee loyalty plays a mediating role between PWG and employee innovative behavior, and OT moderates the conduction mechanism. Therefore, the following hypotheses are proposed:

*H5*: The higher the OT, the stronger PWG affects the employee innovative behavior through employee loyalty.

**Figure 1:**
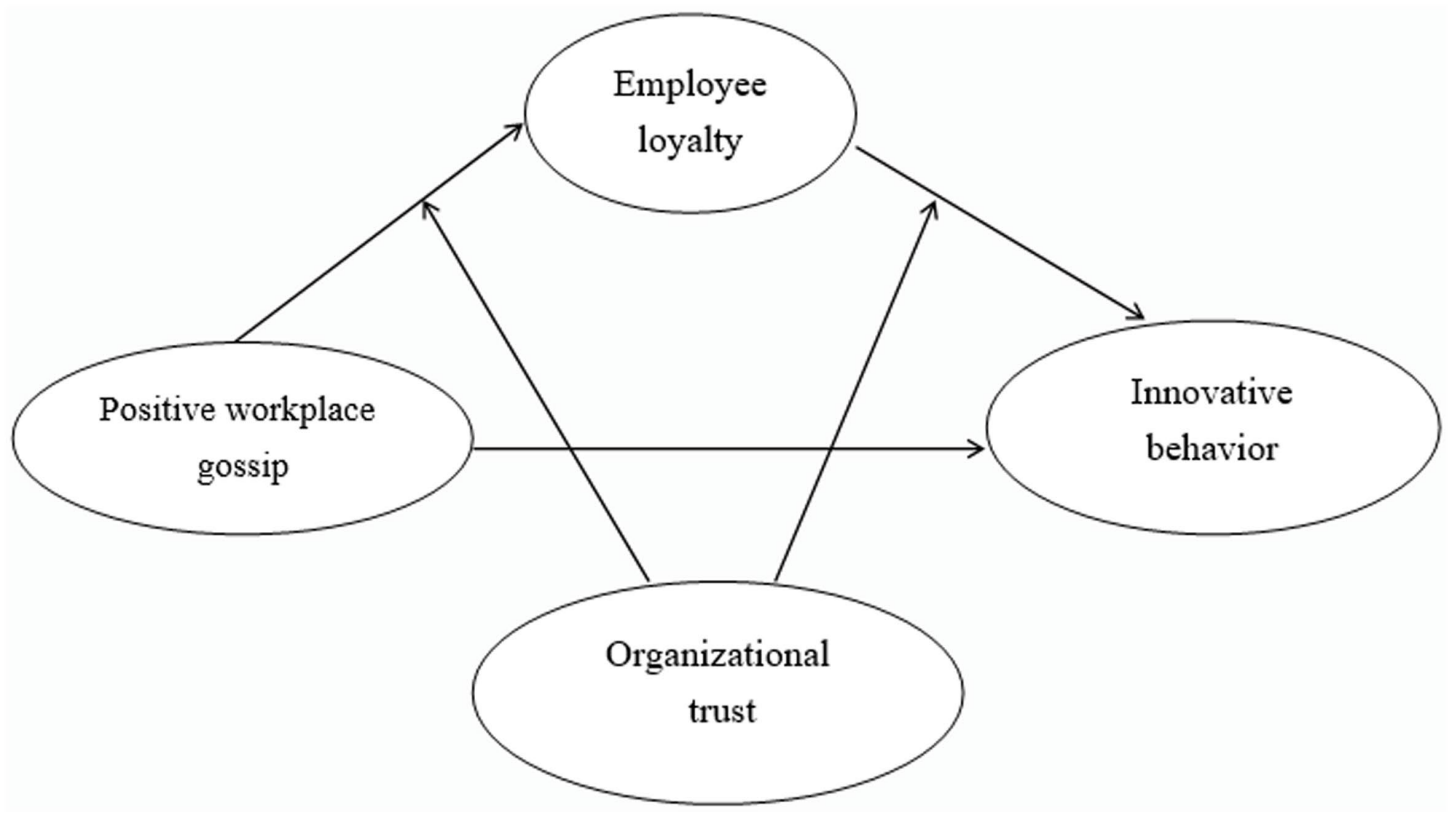
Hypothesized model.

Based on the above theoretical groundwork, this study built the following model as shown in Figure 1.

## Methodology

### Sample and data collection

This study adopted the questionnaire survey method to attain the research objective. The sample enterprises were selected from the Pearl River Delta area and the Yangtze River Delta area of China. Gathering 23.64% of the Chinese population with 4.31% of the national territorial area, both Deltas created 34.65% of China’s economic aggregate (The data originated from China Statistical Yearbook). As important economic hinterlands of China and high-tech industry clustered areas and sci-tech innovation demonstration places confronted with intensely competitive environments, where innovative behaviors are highly required of employees, the Pearl River Delta and the Yangtze River Delta are quite suitable areas for the research. By dint of the advantage that the team members in this study had the membership of the local chamber of commerce, the efficiency of data collection was greatly boosted. To reduce the common method bias (CMB), this study complied with the suggestions of Podsakoff et al. (2003) by separating the data collection at two points-in-time with a 1-month interval in between.

First, the research team made active contact with the corporate persons-in-charge, communicating the matters on questionnaire survey, informing them of the research objective and data collection method, and proclaiming that the survey data were to be used for academic research only. Subsequently, contingent on the condition of the enterprises involved, we handed over the pre-coded questionnaires to each corporate person-in-charge, entrusting him/her to tell his/her employees to fill out whenever appropriate and remind his/her employees to memorize their own number. To reduce the employees’ scruple, we assured the respondents that the information is confidential and that the answers would be made accessible only to the first author and the corresponding author. In the first round, a total of 450 questionnaires were issued, including question items on demographic information, PWG, and organizational trust, and 368 valid questionnaires were recovered (valid recovery rate 81.8%) from 48 corporate teams. On month later, the 368 questionnaires from the first round were filled out for the second round, including question items on employee loyalty and employee innovative behavior, and 327 valid questionnaires were recovered (88.9%) eventually. We paid remunerations to improve the participants’ enthusiasm and control the quality of questionnaire filling. To be specific, we sent a red packet of RMB 10–50 in cash to each respondent in the first round of questionnaire filling.

The industries in this regional survey were distributed mainly in manufacturing, high and new technology, financing, Internet, etc. Among the sampled under investigation, females (57.2%) outnumbered males (42.8%); in terms of age, the age groups of 21–30 years old and 31–40 years old predominated, accounting for 47.2% and 25.1%, respectively, most being front-line employees. Evidently, youngsters were the backbone force of enterprises in these areas; in terms of educational degree, respondents with academic credentials above undergraduate predominated, accounting for 68%; in terms of income level, most had an income of RMB 3,001–5,000 and RMB 5,001–10,000, accounting for 18% and 33%, respectively.

### Measures

All measurement scales selected in this investigation were widely used with good reliability and validity. Meanwhile, to ensure the local applicability of the scales, the question items of each scale were decided on by means of two-way translation, and experts were invited to assess the rigorousness and appropriateness of the questionnaires (Brislin 1970). All constructs in the measures were rated by participants on a 5-point Likert-type scale ranging from strongly disagree (1) to strongly agree (5).

#### Positive workplace gossip

The scale compiled by Brady et al. (2017) was adopted for *PWG*. This scale consisted of 5 questions in total, such as “I have had the deed of complimenting another colleague while talking with a colleague” and “I have spoken high of another colleague in front of one colleague,” and the coefficient of its internal consistency was 0.890 in this investigation.

#### Employee loyalty

In this investigation, the scale used by Matzler and Renzl (2006) in their study was adopted for *employee loyalty*. It consisted of five questions in total, such as “I speak positively about my company when talking to customers” and “I would not change immediately to another company if I got a job offer.” The coefficient of internal consistency of this scale was 0.863 in this investigation.

#### Employee’s innovative behavior

Employee innovative behavior was measured with Scott and Bruce’s (1994) six-item scale. Sample questions included “At work, I come up with innovative and creative notions” and “At work, I seek new service techniques, or methods.” The Cronbach’s alpha for this scale was 0.906.

#### Organizational trust

In this investigation, the scale used by McAllister (1995) in their study was adopted for OT. We made appropriate revisions to this scale and adopted 6 items of it. The Cronbach’s alpha for this scale was 0.929.

#### Control variables

Previous studies demonstrated that gender and educational degree could make a difference to individual innovative behavior, and that individual attitude toward gossip would change with the increase in age (Wu et al., 2018; Eckhaus et al., 2019). Therefore, this investigation took respondent’s *gender*, *age*, and *educational degree* as the control variables. These were denoted in coded forms as *gender*: “1” for male and “2” for female; age: “1” for 20 years old and below, “2” for 21–30 years old, “3” for 31–40 years old, “4” for 41–50 years old, and “5” for 51 years old and above; *educational degree*: “1” for senior high school and below, “2” for junior college, “3” for university undergraduate, and “4” for postgraduate and above.

## Analysis and results

This study adopted the structural equation software Amos24 for confirmatory factor analysis (CFA), conducted reliability test, validity test, and correlation analysis by the software SPSS24, and completed hypothesis verification using the program Process developed by Hayes.

### Reliability and validity tests on research variables

SPSS24 was employed to make reliability analysis on all variables. The Cronbach’s alpha was >0.8 for all measurement variables. In the hypothetical model the factor loading between the measurement questions and latent variables ranged within 0.573–0.845 (greater than the recommended value 0.5), the average variance extracted (AVE) ranged within 0.520–0.685 (greater than the recommended value 0.5), and the composite reliability (CR) of latent variables ranged within 0.842–0.924 (greater than the recommended value 0.7; Fornell and Larcker 1981), indicating that the variables have high internal consistency (see Table 1).

**Table 1:** Factor loadings of variables and overall reliability.

Variables	Items	Factor loadings	Cronbach’s alpha	Composite reliability (CR)	Average variance extracted (AVE)
PWG	PWG1	0.810	0.890	0.916	0.685
	PWG2	0.835			
	PWG3	0.835			
	PWG4	0.833			
	PWG5	0.826			
OT	OT1	0.808	0.929	0.924	0.670
	OT2	0.832			
	OT3	0.811			
	OT4	0.845			
	OT5	0.830			
	OT6	0.784			
EL	EL1	0.762	0.863	0.842	0.520
	EL2	0.749			
	EL3	0.804			
	EL4	0.694			
	EL5	0.573			
EIB	EIB1	0.765	0.906	0.902	0.605
	EIB2	0.789			
	EIB3	0.785			
	EIB4	0.794			
	EIB5	0.779			
	EIB6	0.754			

PWG, positive workplace gossip; OT, Organizational trust; EL, employee loyalty; EIB, Employee innovative behavior.

Next, Amos24 was adopted to conduct CFA on the four variables to examine the model’s discriminant validity. CFA results are shown in Table 2. The four-factor model has the highest goodness of fit (*χ*^2^ = 404.248, *χ*^2^/df = 1.982, GFI = 0.899, CFI = 0.958, RMSEA = 0.055, TLI = 0.953, IFI = 0.958) and is superior to any other alternative model, and the originally designed model has superior discriminant validity.

**Table 2:** Comparison of measurement model.

Models	Factors	*χ* ^2^	*χ*^2^/df	GFI	CFI	RMSEA	TLI	IFI
Baseline Model	Four factors: PWG, OT, EL, EIB	404.248***	1.982	0.899	0.958	0.055	0.953	0.958
Model 1	Three factors: PWG + OT, EL, EIB	1288.072***	6.223	0.665	0.773	0.127	0.746	0.774
Model 2	Three factors: EL + IB, PWG, OT	919.215***	4.441	0.765	0.765	0.103	0.833	0.851
Model 3	Two factors: PWG + OT, EL + EIB	1800.574***	8.615	0.591	0.665	0.153	0.63	0.667
Model 4	Two factors: PWG + OT + EL, EIB	1711.242***	8.227	0.571	0.684	0.149	0.649	0.685
Model 5	One factors: PWG + OT + EL + EIB	2373.619***	11.357	0.471	0.545	0.178	0.497	0.547

PWG, positive workplace gossip; OT, Organizational trust; EL, employee loyalty; EIB, Employee innovative behavior. ***p < 0.001.

### CMB analysis

All the data collected in this investigation were acquired by self-evaluation means, and the empirical results may be subject to CMB. In this study, the Harman single-factor test was used to assess CMB (Podsakoff et al., 2003). The results indicated that there was a total of four factors with characteristic roots >1 extracted from the unrotated exploratory factor analysis (EFA) results; the maximum variation of the interpretation of factors was 40.905% (under the 50% threshold), showing that the effect of CMB on this study is minor.

### Descriptive statistics

Pearson’s coefficient was utilized to analyze the correlation between variables. Table 3 presents the mean, standard deviation, and correlation coefficients between variables. As shown in Table 3, PWG bears a significantly positive correlation to employee innovative behavior (*r* = 0.397, *p* < 0.01); PWG bears a significantly positive correlation to employee loyalty (*r* = 0.418, *p* < 0.01); employee loyalty bears a positive correlation to employee innovative behavior (*r* = 0.560, *p* < 0.01). These results lend preliminary support to the hypotheses proposed in this study and lay a groundwork for the follow-up test.

**Table 3:** Descriptive statistics and correlations among variables.

Variables	Mean	SD	1	2	3	4
1 PWG	3.511	0.841	1			
2 OT	3.498	0.828	0.315**	1		
3 EL	3.636	0.766	0.418**	0.540**	1	
4 EIB	3.645	0.765	0.397**	0.410**	0.560**	1

PWG, positive workplace gossip; OT, Organizational trust; EL, employee loyalty; EIB, Employee innovative behavior. **p < 0.01.

### Testing of hypotheses

First, we adopted the method proposed by (Preacher et al., 2007) to test the mediating effect, using the model 4 in the macro-program SPSS Process, setting 5,000 times of repeated sampling and 95% confidence level. With *gender*, *age*, and *educational degree* controlled, we tested the mediating effects of employee loyalty and PWG on employee innovative behavior. The results are shown in Table 4. PWG has a significant predicative effect on employee innovative behavior (*B* = 0.365, *t* = 7.647, *p* < 0.001), *H1* is verified. After the mediating variable was introduced on this base, the predictive effect of PWG on employee innovative behavior remained significant (*B* = 0.182, *t* = 3.919, *p* < 0.001); PWG has a significant predicative effect on employee loyalty (*B* = 0.382, *t* = 8.155, *p* < 0.001); employee loyalty has a positive significant effect on employee innovative behavior (*B* = 0.481, *t* = 9.581, *p* < 0.001). Additionally, neither the bootstrap95% confidence interval of the direct effect of PWG on employee innovative behavior nor that of the mediating effect of employee loyalty contains 0 between the upper and lower limits, suggesting that PWG can predict employee innovative behavior not only directly but also *via* employee loyalty, *H2* is also corroborated (see Table 5).

**Table 4:** Results on the mediating role of employee loyalty with PWG and employee innovative behavior.

Outcome variable	Predictor	*R*	*R* ^2^	*F*-value	*B*	*t*-value
EIB		0.400	0.160	15.354***		
	Gender				−0.044	−0.546
	Age				0.024	0.589
	Education				0.001	0.020
	PWG				0.365	7.647***
EL		0.440	0.193	19.309***		
	Gender				−0.093	−1.188
	Age				0.101	2.480
	Education				0.030	0.697
	PWG				0.382	8.155***
EIB		0.668	0.446	43.012***		
	Gender				0.001	0.016
	Age				−0.024	−0.651
	Education				−0.014	−0.350
	PWG				0.182	3.919***
	EL				0.481	9.581***

PWG, positive workplace gossip; OT, Organizational trust; EL, employee loyalty; EIB, Employee innovative behavior. *p < 0.05, **p < 0.01, ***p < 0.001.

**Table 5:** Results of indirect effects, direct effects, and total effects.

	Effect	Boot SE	Boot LL CI	Boot ULCI	%
Indirect effect	0.183	0.034	0.120	0.252	50.428%
Direct effect	0.180	0.052	0.080	0.286	49.572%
Total effect	0.362	0.052	0.257	0.462	

CI, confidence interval; LL, lower limit; UL, upper limit. Bootstrap sample size = 5,000.

**Figure 2:**
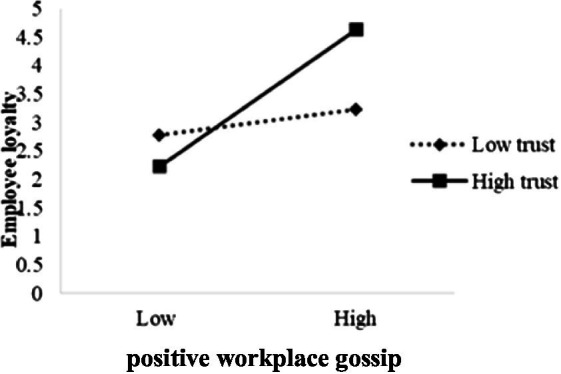
The interaction between positive workplace gossip and organizational trust on employee loyalty.

**Figure 3:**
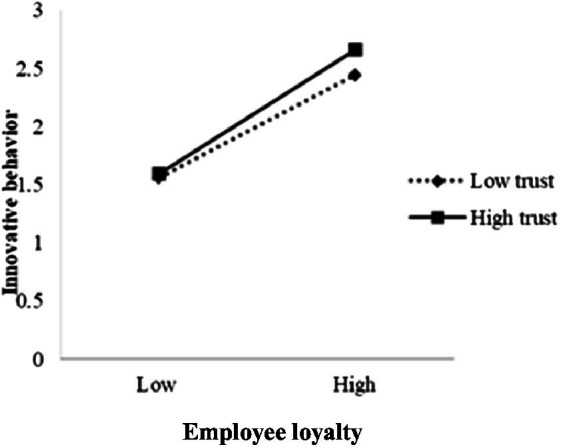
The interaction between employee loyalty and organizational trust on employee innovative behavior.

Next, the test procedure of conditional indirect effect proposed by (Preacher et al., 2007) was adopted, and the model in the SPSS macro compiled by Hayes corresponding to this study was selected. With *gender*, *age*, and *educational degree* controlled, the mediator model with regulation was tested. The results (see Table 6) show that, after putting OT into the model, the product term of PWG and intraorganizational trust has a significant predictive effect on employee loyalty (*B* = 0.098, *t* = 2.416, *p* < 0.01), so does the product term of employee loyalty and intraorganizational trust on employee innovative behavior (*B* = 0.097, *t* = 1.945, *p* < 0.05), suggesting that intraorganizational trust can act a mediating role in the predictions of PWG about employee loyalty and of employee loyalty about employee innovative behavior. Hence, *H3* and *H4* are verified. Through further simple slope analysis, the positive influential effect of PWG on employee loyalty is strengthened under the circumstance of high organizational trust (+1 SD) compared to low organizational trust (−1 SD; see Figure 2); the positive influential effect of employee loyalty on employee innovative behavior is strengthened under the circumstance of high organizational trust (+1 SD) compared to low organizational trust (−1 SD; see Figure 3).

**Table 6:** Test of moderating effects.

Output variable	Predictor	*R*	*R* ^2^	*F*	*B*	*t*-value
EL		0.638	0.407	36.945^***^		
	Gender				−0.057	−0.846
	Age				0.148	4.235^***^
	Education				0.076	2.03
	PWG				0.225	5.314^***^
	OT				0.098	10.252^***^
	PWG × OT				0.098	2.416^**^
EIB		0.604	0.365	23.093^***^		
	Gender				0.006	0.087
	Age				0.000	0.003
	Education				0.002	0.058
	EL				0.439	7.472^***^
	OT				0.126	2.481^**^
	EL × OT				0.097	1.945^*^

PWG, positive workplace gossip; OT, organizational trust; EL, employee loyalty; EIB, employee innovative behavior. *p < 0.05, **p < 0.01, ***p < 0.001.

In addition, to test the moderated mediation relationship predicted by *H5,* we examined various conditional indirect effects of PWG on employee innovative behavior, *via* employee loyalty, across high and low levels of organizational trust using the PROCESS program of Hayes. As proposed by (Preacher et al., 2007), we operationalized high and low levels of organizational trust as 1 standard deviation above and below the variable’s mean score. Bootstrapping results revealed that the conditional indirect effect of PWG on employee innovative behavior was stronger with high organizational trust (effect = 0.150, 95% CI [0.087, 0.215]) but weaker and not significant with low organizational trust (effect = 0.053, 95% CI [−0.006, 0.125]; see Table 7).

**Table 7:** Moderated mediation effect test.

Moderator	Level	Conditional indirect effect	Boot SE	95% CI	
				LL	UL
Organizational	Low (mean − 1 SD)	0.053	0.033	−0.006	0.125
trust	High (mean + 1 SD)	0.150	0.032	0.087	0.215

SD, standard deviation; CI, confidence interval; LL, lower limit; UL, upper limit. Bootstrap sample size = 5,000.

## Discussion

The mechanism of action on employee innovative behavior is complicated. Employee innovative behavior is subject not only to job characteristics and individual factors but also to environmental factors. Gossip is a ubiquitous phenomenon in various social environments (Foster 2004; Estévez et al., 2022), and is considered an important nonformal way by which people acquire information and emotional connection in an environment with uncertainties (Wert and Salovey 2004). In recent years, workplace gossip has been studied in different fields (Wu et al., 2018; Bencsik and Juhasz 2020; Lee and Barnes 2021). Compared with predecessors’ studies, the research outcomes of workplace gossip and employee’s innovative behavior are scant and mainly concentrated on the negative influences (Zhou et al., 2019). For example, the findings of Liu et al. (2020) from 451 Chinese employees and managers suggested employees perceiving of negative gossip tended to experience emotional exhaustion, thereby weakening their creativity. Researchers are zealous for the study on negative gossip while overlooking the function of positive gossip (Wu et al., 2018). The main reason is that negative gossip has a negative influence on gossip participants and the organization and a stronger destructive power (Wu et al., 2018), while the effect of positive gossip is limited mainly to promoting interpersonal relationships and information exchange. But it turns out from this study that employees circulating positive workplace gossip tend to exhibit more innovative behaviors in the work. Gossip is opined as a tool to understand a socializing environment (Aghbolagh et al., 2021). We argue that there are at least two sides of positive effects of employees circulating positive gossip in workplace: On the one hand, positive gossip can create a harmonious socializing environment, which is favorable for shaping a good innovation atmosphere; on the other hand, positive gossip is conducive to integrating employees into the workplace circle and developing a good sense of belonging to the organization. These positive effects can promote employee innovative behavior.

After introducing employee loyalty as a mediating variable in this study, it was found that PWG has both direct and indirect effects on employee innovative behavior. PWG is a tie *via* which to build a friendship between employees (Ellwardt et al., 2012b), and an effective means to promote teamwork and job satisfaction. Previous studies found that job satisfaction would make a difference to employees’ working behavior, attitude, and organizational performance *via* the mediating role of employee loyalty (Phuong and Vinh 2020; Zanabazar and Jigjiddorj 2021). This study has arrived at the similar conclusion. In the past, researchers believed that negative workplace gossip usually carried pejorative and denigrative intentions, and that it was a workplace behavior to be attacked and repelled for it could affect employees’ physical and mental health (Beersma and Van Kleef 2012). Therefore, many researchers deemed negative workplace gossip as a “cold violence in workplace.” While our research findings have substantiated that positive gossip can not only promote employee loyalty but also indirectly affect innovative behavior *via* employee loyalty. Therefore, we believe positive gossip functions like a “positive-energy catalyst” in helping promote interpersonal relationships in workplace and positively affecting employee loyalty and innovative behavior.

Concerning the relation between organizational trust and workplace gossip, Ellwardt et al. (2012c) believed that the gossip communication speed was constant whether in a low-trust or in a high-trust environment. They also noted that, relative to negative gossip, positive gossip was less risky and therefore less susceptible to trust. However, our research findings have arrived at the opposite conclusion, confirming that the level of organizational trust can affect PWG. To be specific, the higher the level of organizational trust, the stronger the positive effect of PWG on employee loyalty. This is because in a high-level organizational trust environment, employees are more ready to share information, hence more willing and likely to communicate positive gossip, and thus the effect of PWG on employee loyalty is strong. Furthermore, this study has corroborated that PWG can regulate the positive effects on employee loyalty and employee innovative behavior. Trust can ameliorate the relationship between innovative teams. Nowadays, the new generation of employees have raised higher requirement on workplace, not just pursuing material demands but also valuing internal emotional needs. The innovative impetus of an employee who has long been working in an environment lacking in trust will inevitably be hampered. At a high-trust level, organization members are more ready to exchange their ideas and share their knowledge, and innovators having a sense of loyalty are even more ready to unleash their innovative thinking and exhibit more innovative capabilities.

## Conclusion

Keeping a foothold on China’s situation, this study has designed a moderated mediation model from the gossip sender’s perspective and examined the effects of positive gossip by empirical method. The results have shown that PWG has a significantly positive influence on employee innovative behavior, and that employee loyalty mediates the PWG-employee innovative behavior relation; OT can moderate PWG’s indirect influence on employee innovative behavior through employee loyalty. The above research findings are of enlightening significance in both theory and management practice.

### Theoretical contributions

First, this research has promoted the research in the field of employee innovative behavior. Over the past decade, in mass studies on innovation, scholars were particularly concentrated on the antecedent variables affecting innovation and their consequent variables (Farrukh et al., 2022). Organizational situational factors are important ones influencing employee innovative behavior. As one such key situational factor affecting interpersonal relationships, workplace gossip has been widely confirmed to affect employees’ behavior (e.g., Spoelma and Hetrick 2021; Zong et al., 2021). Although gossip is ubiquitous in workplace, almost studies on gossip sender’s influence thus far have been concentrated on the influence of negative gossip (Zhou et al., 2019). This study has explored the mediating mechanism and boundary condition of PWG on employee innovative behavior, providing a beneficial supplement for previous studies on employee innovative behavior.

Second, this study has introduced employee loyalty to disclose the indirect effect amid the influence of PWG on employee innovative behavior. The result of the research expands the influencing factors of employee loyalty. Previous studies show that scholars have done a lot of research on the antecedent variables of employee loyalty, which include various factors such as the individual, the organization, and environment (Guillon and Cezanne 2014; Nadeak and Naibaho 2020), but few studies address the impact of workplace gossip on employee loyalty. This paper takes employee loyalty as a key process in which PWG influences employee innovation behavior, and proves its existence and significance. Therefore, this study extends existing knowledge of employee loyalty.

Third, this study broadens the PWG boundary condition theory by identifying organizational trust as a moderating factor. Tan et al. (2021) advised that research on workplace gossip should closely examine the emotional attachments and trusting relationship, as this might have a significant impact on how employees respond to gossip. This study answers their call and reveals that organizational trust appears to be a crucial boundary condition for positive workplace gossip. Furthermore, whether PWG or organizational trust is a situational factor of workplace. This study integrated both together to examine their influence upon employee innovative behavior, supplementing the studies on the influence of multiple situational factors upon employee innovative behavior.

### Managerial implications

Developing personal relationships beyond workplace plays a significant role in employees’ working behavior and interpersonal relationship building. Existing studies on the consequence of gossip have arrived at conflicting viewpoints. Some researchers believe that gossip is detrimental, while some scholars believe it is beneficial. These contradictory results have made managers feel helpless in dealing with the problem of gossip (Dores Cruz et al., 2021). This study focuses on the influence of PWG on employee innovative behavior. The research findings are of several significances, as follows, to management practice:

First, the research findings have indicated PWG has a significantly positive effect on employee innovative behavior. Considering this, organizations should pay more attention to workplace gossip phenomena, encourage employees to take appropriate part in positive workplace gossip (Zong et al., 2021) and to communicate positive information about other colleagues frequently, and build an inclusive, open, sincere, and interdependent platform in the organization, not only for exchanging information and building good interpersonal relationships but also for gaining workplace friendship and meeting emotional needs, thereby improving employee innovative behavior. On the other hand, while communicating positive gossip, employees can learn the positive aspects from the gossip target as a good example, and motivate themselves to work hard and unleash their innovative potential to a higher level in the work.

Next, how to retain employees and build employee loyalty have become the issues concerned by global human resources managers nowadays (Dutta and Dhir 2021). Managers usually take such measures as promotion, pay hike, and welfare increase to raise employee loyalty (Nadeak and Naibaho 2020). The findings of this study have shown that PWG has a significantly positive effect on employee loyalty. Therefore, to attract and retain high-quality, dedicated, high-loyalty employees, enterprises should create a wholesome working environment for them (Dhir et al., 2020) and positively encourage employees to communicate positive workplace gossips, so as to create a harmonious workplace atmosphere and raise employee loyalty.

Lastly, organizational trust can significantly regulate the strength of the effects of PWG on employee loyalty and of employee loyalty on employee innovative behavior. Therefore, enterprises should pay attention to enhancing the factors for employees’ trust in the organization, emphasize constructing a communicative, transparent, and inclusive cultural environment (Paşamehmetoğlu et al., 2022). Managers can also build a good superior-subordinate relationship by means of authorization, guidance, care, and encouragement, etc., encourage employees to express their ideas freely, and give timely feedback of employees’ needs. These attempts will enhance employees’ trust in the managers and the organization (Berraies et al., 2021), raise employees’ sense of security, and promote employee innovative behavior.

### Limitations and future direction

Although this study has drawn some important conclusions from the investigation into the influence of PWG on employee innovative behavior, there remain some limitations and deficiencies, as manifested mainly in the following aspects. First, different cultural backgrounds would give rise to different employee behaviors. All survey samples in this study coming from the Pearl River Delta area and the Yangtze River Delta area of Chinese mainland, the research findings have certain limitations in general applicability. Future studies can make a supplement from the perspective of multicultural background. Second, all data in this study stemmed from employees’ self-evaluation. Although the same-source variance of data was tested after the investigation, and the results were in a reasonable range, the problem of same-source variance could not be eradicated. To ensure higher preciseness of data, the data sources in future studies can be compensated for *via* research and design by means of employee evaluation and leader evaluation. Third, the study on employee innovative behavior is a complicated project. This paper has only explored the influence of PWG on employee innovative behavior, and the antecedent variables and influencing mechanism remain to be explored and enriched. For instance, future studies can further discuss the influence of negative workplace gossip (NWG) on employee innovative behavior.

## Data availability statement

The original contributions presented in the study are included in the article/supplementary material, further inquiries can be directed to the corresponding author.

## Author contributions

Material preparation, data collection, and analysis were performed by YD, XZ, JH, and BL. The first draft of the manuscript was written by YD. All authors contributed to the study conception, design, and commented on previous versions of the manuscript. All authors read and approved the final manuscript.

## Funding

This work was supported by the National Social Science Foundation (21FYYB056), and JH (houjie918@163.com), School of Foreign Languages, Huaibei Normal University (235000).

## Conflict of interest

The authors declare that the research was conducted in the absence of any commercial or financial relationships that could be construed as a potential conflict of interest.

## Publisher’s note

All claims expressed in this article are solely those of the authors and do not necessarily represent those of their affiliated organizations, or those of the publisher, the editors and the reviewers. Any product that may be evaluated in this article, or claim that may be made by its manufacturer, is not guaranteed or endorsed by the publisher.

## References

[ref1] Aghbolagh M. B. Ardabili F. S. Voitenko E. (2021). Content analysis of gossip at different levels of a hospital. Organ 54, 306–318. doi: 10.2478/orga-2021-0021

[ref2] Alshaabani A. Hamza K. A. Rudnák I. (2022). Impact of diversity management on employees’ engagement: the role of organizational trust and job insecurity. Sustainability 14:420. doi: 10.3390/su14010420

[ref3] Amabile T. M. Pratt M. G. (2016). The dynamic componential model of creativity and innovation in organizations: making progress, making meaning. Res. Organ. Behav. 36, 157–183. doi: 10.1016/j.riob.2016.10.001

[ref4] Beehner C. G. Blackwell M. J. (2016). The impact of workplace spirituality on food service worker turnover intention. J. Manage. Spiritual. Relig. 13, 304–323. doi: 10.1080/14766086.2016.1172251

[ref5] Beersma B. Van Kleef G. A. (2012). Why people gossip: an empirical analysis of social motives, antecedents, and consequences. J. Appl. Soc. Psychol. 42, 2640–2670. doi: 10.1111/j.1559-1816.2012.00956.x

[ref6] Bencsik A. Juhasz T. (2020). Impacts of informal knowledge sharing (workplace gossip) on organisational trust. Econ. Soc. 13, 249–270. doi: 10.14254/2071-789x.2020/13-1/16

[ref001] Berraies S. Hamza K. A. Chtioui R. (2021). Distributed leadership and exploratory and exploitative innovations: mediating roles of tacit and explicit knowledge sharing and organizational trust. J. Knowl. Manag. 25, 1287–1318. doi: 10.1108/JKM-04-2020-0311, PMID: 27732002

[ref7] Brady D. L. Brown D. J. Liang L. H. (2017). Moving beyond assumptions of deviance: the reconceptualization and measurement of workplace gossip. J. Appl. Psychol. 102, 1–25. doi: 10.1037/apl0000164, PMID: 27732002

[ref8] Brislin R. W. (1970). Back-translation for cross-cultural research. J. Cross-Cult. Psychol. 1, 185–216. doi: 10.1177/135910457000100301

[ref006] Chen L. M. Guo Y. R. Song L. J. Lyu B. (2020). From errors to OCBs and creativity: a multilevel mediation mechanism of workplace gratitude. Curr. Psychol. 41, 6170–6184. doi: 10.1007/s12144-020-01120-5

[ref9] Cropanzano R. Mitchell M. S. (2005). Social exchange theory: an interdisciplinary review. J. Manag. 31, 874–900. doi: 10.1177/0149206305279602

[ref10] Dhir S. Dutta T. Ghosh P. (2020). Linking employee loyalty with job satisfaction using PLS–SEM modelling. Pers. Rev. 49, 1695–1711. doi: 10.1108/PR-03-2019-0107

[ref11] Dores Cruz T. D. Nieper A. S. Testori M. Martinescu E. Beersma B. (2021). An integrative definition and framework to study gossip. Group Organ. Manage. 46, 252–285. doi: 10.1177/1059601121992887

[ref12] Dutta T. Dhir S. (2021). Employee loyalty: measurement and validation. Glob. Bus. Rev. 1–18. doi: 10.1177/0972150921990809

[ref13] Eckhaus E. Ben-Hador B. J. J. O G. S. (2019). Gossip and gender differences: a content analysis approach. J. Gend. Stud. 28, 97–108. doi: 10.1080/09589236.2017.1411789

[ref14] Eisenberger R. Rhoades L. (2001). Incremental effects of reward on creativity. J. Pers. Soc. Psychol. 81, 728–741. doi: 10.1037/0022-3514.81.4.728, PMID: 11642357

[ref15] Ellwardt L. Labianca G. Wittek R. (2012a). Who are the objects of positive and negative gossip at work? A social network perspective on workplace gossip. Soc. Networks 34, 193–205. doi: 10.1016/j.socnet.2011.11.003

[ref16] Ellwardt L. Steglich C. Wittek R. (2012b). The co-evolution of gossip and friendship in workplace social networks. Soc. Netw. 34, 623–633. doi: 10.1016/j.socnet.2012.07.002

[ref17] Ellwardt L. Wittek R. Wielers R. (2012c). Talking about the boss: effects of generalized and interpersonal trust on workplace gossip. Group Organ. Manage. 37, 521–549. doi: 10.1177/1059601112450607

[ref18] Estévez J. L. Kisfalusi D. Takács K. (2022). More than one’s negative ties: the role of friends’ antipathies in high school gossip. Soc. Netw. 70, 77–89. doi: 10.1016/j.socnet.2021.11.009

[ref19] Farley S. D. (2011). Is gossip power? The inverse relationships between gossip, power, and likability. Eur. J. Soc. Psychol. 41, 574–579. doi: 10.1002/ejsp.821

[ref20] Farrukh M. Meng F. Raza A. Wu Y. (2022). Innovative work behaviour: the what, where, who, how and when. Pers. Rev. doi: 10.1108/PR-11-2020-0854

[ref21] Fornell C. Larcker D. F. (1981). Evaluating structural equation models with unobservable variables and measurement error. J. Mark. Res. 18, 39–50. doi: 10.1177/002224378101800104

[ref22] Foster E. K. (2004). Research on gossip: taxonomy, methods, and future directions. Rev. Gen. Psychol. 8, 78–99. doi: 10.1016/j.orgdyn.2011.12.007

[ref23] Grosser T. Kidwell V. Labianca G. J. (2012). Hearing it through the grapevine: positive and negative workplace gossip. Organ. Dyn. 41, 52–61. doi: 10.1016/j.orgdyn.2011.12.007

[ref24] Grosser T. J. Lopez-Kidwell V. Labianca G. (2010). A social network analysis of positive and negative gossip in organizational life. Group Org. Manag. 35, 177–212. doi: 10.1177/1059601109360391

[ref25] Guillon O. Cezanne C. (2014). Employee loyalty and organizational performance: a critical survey. J. Organ. Chang. Manag. 27, 839–850. doi: 10.1108/JOCM-02-2014-0025

[ref26] Hsu M. L. Chen F. H. (2017). The cross-level mediating effect of psychological capital on the organizational innovation climate–employee innovative behavior relationship. J. Creat. Behav. 51, 128–139. doi: 10.1002/jocb.90

[ref27] Hui C. H. Pak S. T. Kwan S. O. Chao A. (2012). Attributional style and engagement/disengagement responses in the Chinese workforce. Appl. Psychol. 61, 204–226. doi: 10.1111/j.1464-0597.2011.00463.x

[ref28] Lee S. H. Barnes C. M. (2021). An attributional process model of workplace gossip. J. Appl. Psychol. 106, 300–316. doi: 10.1037/apl0000504, PMID: 32297765

[ref29] Lewis J. D. Weigert A. (1985). Trust as a social reality. Soc. Forces 63, 967–985. doi: 10.1093/sf/63.4.967

[ref30] Liu X.-Y. Kwan H. K. Zhang X. (2020). Introverts maintain creativity: a resource depletion model of negative workplace gossip. Asia Pac. J. Manag. 37, 325–344. doi: 10.1007/s10490-018-9595-7

[ref007] Lyu B. Liao X. Y. Yang Y. C. (2022). Relationships Between Temporal Leadership, Transactive Memory Systems and Team Innovation Performance. Psychol. Res. Behav. Manag. 15, 2543–2559. doi: 10.2147/PRBM.S380989 36124335 PMC9482459

[ref31] Matzler K. Renzl B. (2006). The relationship between interpersonal trust, employee satisfaction, and employee loyalty. Total Qual. Manage. Bus. Excell. 17, 1261–1271. doi: 10.1080/14783360600753653

[ref32] McAllister D. J. (1995). Affect-and cognition-based trust as foundations for interpersonal cooperation in organizations. Acad. Manag. J. 38, 24–59. doi: 10.5465/256727

[ref33] Meyer J. P. Allen N. J. (1991). A three-component conceptualization of organizational commitment. Hum. Resour. Manag. Rev. 1, 61–89. doi: 10.1016/1053-4822(91)90011-Z

[ref34] Mossholder K. W. Settoon R. P. Henagan S. C. (2005). A relational perspective on turnover: examining structural, attitudinal, and behavioral predictors. Acad. Manag. J. 48, 607–618. doi: 10.5465/amj.2005.17843941

[ref35] Nadeak B. Naibaho L. (2020). Motivation and HRM factors relation to the employee loyalty. Pol. J. Manag. Stud. 22, 261–276. doi: 10.17512/pjms.2020.22.2.18

[ref36] Noon M. Delbridge R. (1993). News from behind my hand: gossip in organizations. Organ. Stud. 14, 23–36. doi: 10.1177/017084069301400103

[ref37] Olokundun M. Ibidunni S. Ogbari M. Falola H. Salau O. (2021). COVID-19 pandemic and antecedents for digital transformation in the workplace: a conceptual framework. Open Access Maced. J. Med. Sci. 9, 41–46. doi: 10.3889/oamjms.2021.4952

[ref38] Østergaard C. R. Timmermans B. Kristinsson K. (2011). Does a different view create something new? The effect of employee diversity on innovation. Res. Policy 40, 500–509. doi: 10.1016/j.respol.2010.11.004

[ref002] Paşamehmetoğlu A. Guzzo R. F. Guchait P. (2022). Workplace ostracism: Impact on social capital, organizational trust, and service recovery performance. J. Hosp. Tour. Manag. 50, 119–126. doi: 10.1016/j.jhtm.2022.01.007, PMID: 26821081

[ref39] Phuong T. T. K. Vinh T. T. (2020). Job satisfaction, employee loyalty and job performance in the hospitality industry: a moderated model. Asian Econ. Financ. Rev. 10, 698–713. doi: 10.18488/journal.aefr.2020.106.698.713

[ref40] Podsakoff P. M. MacKenzie S. B. Lee J.-Y. Podsakoff N. P. (2003). Common method biases in behavioral research: a critical review of the literature and recommended remedies. J. Appl. Psychol. 88, 879–903. doi: 10.1037/0021-9010.88.5.879, PMID: 14516251

[ref41] Preacher K. J. Rucker D. D. Hayes A. F. (2007). Addressing moderated mediation hypotheses: theory, methods, and prescriptions. Multivar. Behav. Res. 42, 185–227. doi: 10.1080/00273170701341316, PMID: 26821081

[ref003] Scott S. G. Bruce R. A. (1994). Determinants of innovative behavior: a path model of individual innovation in the workplace. Acad. Manage. J. 37, 580–607. doi: 10.5465/256701

[ref42] Shore L. M. Tetrick L. E. Lynch P. Barksdale K. (2006). Social and economic exchange: construct development and validation. J. Appl. Soc. Psychol. 36, 837–867. doi: 10.1111/j.0021-9029.2006.00046.x

[ref43] Singh U. Srivastava K. B. L. (2016). Organizational trust and organizational citizenship behaviour. Glob. Bus. Rev. 17, 594–609. doi: 10.1177/0972150916630804

[ref44] Spoelma T. M. Hetrick A. L. (2021). More than idle talk: examining the effects of positive and negative team gossip. J. Organ. Behav. 42, 604–618. doi: 10.1002/job.2522

[ref005] Su W. L. Lyu B. Chen H. Zhang Y. Z. (2020). How does servant leadership influence employees’ service innovative behavior? The roles of intrinsic motivation and identification with the leader. Balt. J. Manag. 15, 571–586. doi: 10.1108/BJM-09-2019-0335

[ref004] Tan N. Yam K. C. Zhang P. Brown D. J. (2021). Are you gossiping about me? The costs and benefits of high workplace gossip prevalence. J. Bus. Psychol. 36, 417–434. doi: 10.1007/s10869-020-09683-7

[ref45] Turner J. C. (1975). Social comparison and social identity: some prospects for intergroup behaviour. Eur. J. Soc. Psychol. 5, 1–34. doi: 10.1002/ejsp.2420050102

[ref46] Ugwu F. O. Onyishi I. E. Rodríguez-Sánchez A. M. (2014). Linking organizational trust with employee engagement: the role of psychological empowerment. Pers. Rev. 43, 377–400. doi: 10.1108/PR-11-2012-0198

[ref47] Verburg R. M. Nienaber A.-M. Searle R. H. Weibel A. Den Hartog D. N. Rupp D. E. (2018). The role of organizational control systems in employees’ organizational trust and performance outcomes. Group Organ. Manage. 43, 179–206. doi: 10.1177/1059601117725191, PMID: 29568213 PMC5834078

[ref48] Wert S. R. Salovey P. (2004). A social comparison account of gossip. Rev. Gen. Psychol. 8, 122–137. doi: 10.1037/1089-2680.8.2.122

[ref49] Wu L.-Z. Birtch T. A. Chiang F. F. Zhang H. (2018). Perceptions of negative workplace gossip: a self-consistency theory framework. J. Manag. 44, 1873–1898. doi: 10.1177/0149206316632057

[ref50] Yu M.-C. Mai Q. Tsai S.-B. Dai Y. (2018). An empirical study on the organizational trust, employee-organization relationship and innovative behavior from the integrated perspective of social exchange and organizational sustainability. Sustainability 10:864. doi: 10.3390/su10030864

[ref51] Zaheer A. McEvily B. Perrone V. (1998). Does trust matter? Exploring the effects of interorganizational and interpersonal trust on performance. Organ. Sci. 9, 141–159. doi: 10.1287/orsc.9.2.141

[ref52] Zanabazar A. Jigjiddorj S. (2021). The mediating effect of employee loyalty on the relationship between job satisfaction and organizational performance. J. Ilmiah Peuradeun 9, 467–481. doi: 10.26811/peuradeun.v9i2.530

[ref53] Zhou A. Q. Liu Y. Su X. Xu H. Y. (2019). Gossip fiercer than a tiger: effect of workplace negative gossip on targeted employees' innovative behavior. Soc. Behav. Pers. 47, 1–11. doi: 10.2224/sbp.5727

[ref54] Zong B. Zhang L. Chu X. Qu J. (2021). Does positive workplace gossip help socialize newcomers? A dual-pathway model based on network ties. PsyCh J. 10, 767–776. doi: 10.1002/pchj.468, PMID: 34137195

